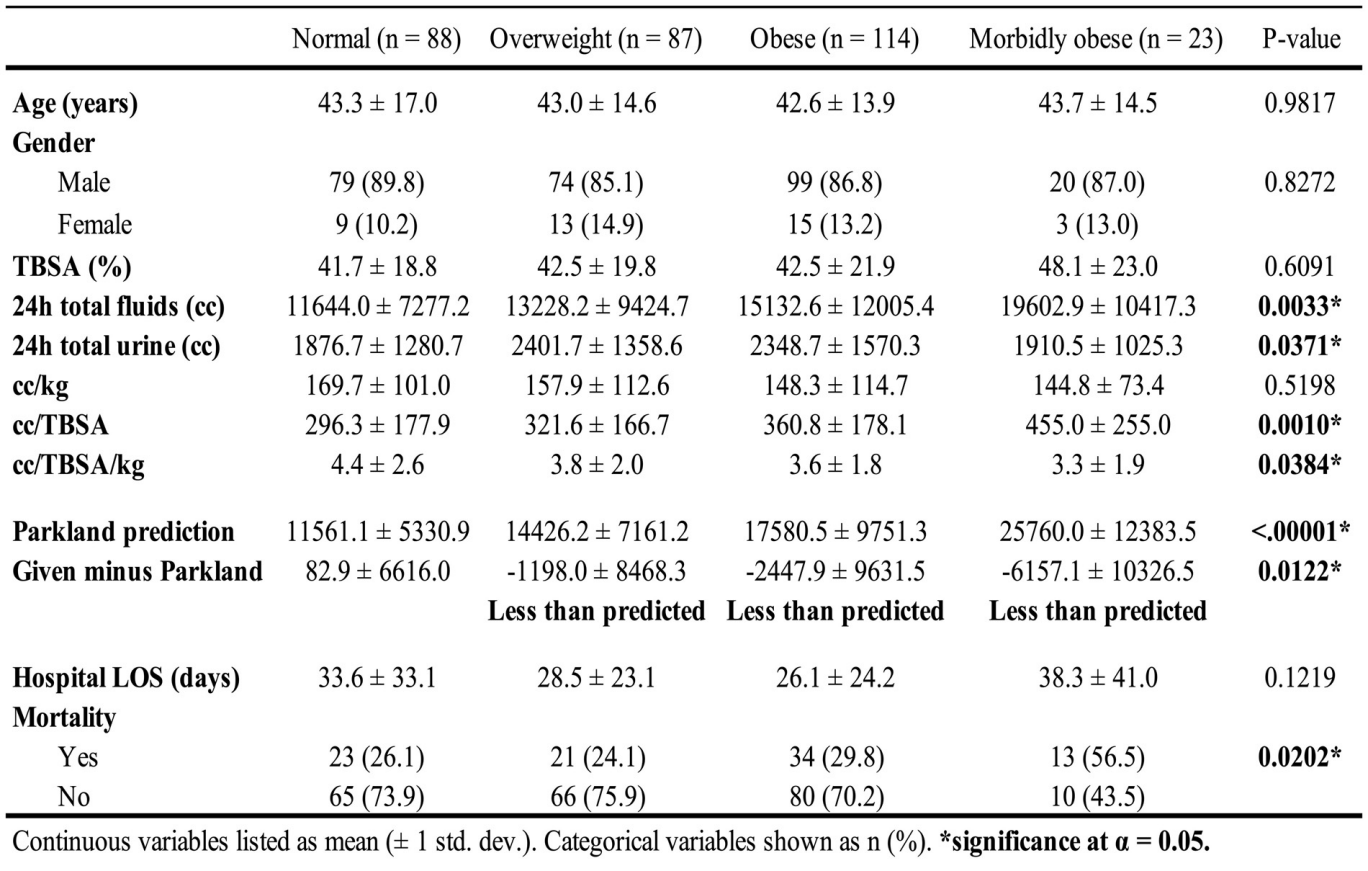# 878 Evaluating the Impacts of Body Mass Index on Fluid Resuscitation and Outcomes in Burn Injuries

**DOI:** 10.1093/jbcr/iraf019.409

**Published:** 2025-04-01

**Authors:** Andrew Ibrahim, Roald Credo, Merry Mathew, Lara Shehadeh, John Griswold

**Affiliations:** Texas Tech University Health Sciences Center School of Medicine; Texas Tech University Health Sciences Center School of Medicine; Texas Tech University Health Sciences Center School of Medicine; Texas Tech University Health Sciences Center School of Medicine; Texas Tech University Health Science Center, Department of Surgery

## Abstract

**Introduction:**

In the management of burn injuries, accurate fluid resuscitation plays a critical role in restoring intravascular volume and avoiding fluid overload complications, which can escalate mortality and morbidity significantly. Protocols such as the 1960s Parkland formula have reduced such complications, yet differing body mass indices (BMI) in burn patients pose physiological impacts that may not be accounted for by these formulas. This study investigates the influence of BMI on the volume of fluids administered in the first 24 hours of burn treatment, comparing actual resuscitation volumes to those predicted by the Parkland formula. This approach aims to address the paucity of literature on optimal fluid resuscitation for obese burn patients, improving patient outcomes in a demographic that has significantly grown over the past decades.

**Methods:**

A retrospective chart review was conducted at a level I trauma center with a dedicated burn unit. The study reviewed records from January 2010 to January 2022 to identify patients aged 18 to 75 years who received treatment for burns >20% total body surface area (TBSA).

**Results:**

This 12-year retrospective study evaluated 312 burn patients categorized by BMI: Normal (n = 88), Overweight (n = 87), Obese (n = 114), and Morbidly Obese (n = 23). Age and gender across BMI groups showed no significant differences (p = 0.982, p = 0.827). TBSA was also not significantly different (p = 0.6091).

Fluid resuscitation showed significant variations across BMI groups. Both total fluids administered and total urine output in the first 24 hours increased as weight groups increased (p = 0.003, p = 0.037). Fluid given per TBSA (cc/TBSA) increased with higher BMI (p = 0.001), and fluid given per TBSA/kg (cc/TBSA/kg) decreased with higher BMI (p = 0.038). Administered fluid volumes were significantly less than those predicted by the Parkland formula in overweight, obese, and especially morbidly obese patients, and deviations increased with higher BMI (p= 0.012). Mortality significantly differed across BMI groups, with highest rates found in morbidly obese patients (p = 0.020).

**Conclusions:**

These findings suggest a multifaceted impact of weight on fluid resuscitation, first shown by the increase in fluids required per TBSA as BMI increases. Additionally, administered fluid volumes were lower than those predicted by the Parkland formula in overweight, obese, and morbidly obese patients, overestimated by 1.2L, 2.4L, and 6.2L, respectively. This suggests that the use of body weight may result in over-resuscitation in higher BMIs, and rates must be adjusted frequently to respond to fluid responsiveness.

**Applicability of Research to Practice:**

In the context of a rising average BMI in the patient population and higher mortality observed in morbidly obese burn patients, these results advocate for the constant management and reevaluation of fluid resuscitation in higher BMI patients to minimize risk of fluid overload and ensure optimal restoration of volume.

**Funding for the Study:**

N/A